# SUMOylation indirectly suppresses activity of the HIF-1α pathway in intestinal epithelial cells

**DOI:** 10.1016/j.jbc.2023.105280

**Published:** 2023-09-22

**Authors:** Mykyta I. Malkov, Darragh Flood, Cormac T. Taylor

**Affiliations:** Conway Institute of Biomolecular and Biomedical Research and School of Medicine, University College Dublin, Belfield, Ireland

**Keywords:** hypoxia, hypoxia-inducible factor, small ubiquitin-like modifier, sumoylation, intestinal epithelium

## Abstract

The hypoxia-inducible factor (HIF) is a master regulator of the cellular transcriptional response to hypoxia. While the oxygen-sensitive regulation of HIF-1α subunit stability *via* the ubiquitin–proteasome pathway has been well described, less is known about how other oxygen-independent post-translational modifications impact the HIF pathway. SUMOylation, the attachment of SUMO (small ubiquitin-like modifier) proteins to a target protein, regulates the HIF pathway, although the impact of SUMO on HIF activity remains controversial. Here, we examined the effects of SUMOylation on the expression pattern of HIF-1α in response to pan-hydroxylase inhibitor dimethyloxalylglycine (DMOG) in intestinal epithelial cells. We evaluated the effects of SUMO-1, SUMO-2, and SUMO-3 overexpression and inhibition of SUMOylation using a novel selective inhibitor of the SUMO pathway, TAK-981, on the sensitivity of HIF-1α in Caco-2 intestinal epithelial cells. Our findings demonstrate that treatment with TAK-981 decreases global SUMO-1 and SUMO-2/3 modification and enhances HIF-1α protein levels, whereas SUMO-1 and SUMO-2/3 overexpression results in decreased HIF-1α protein levels in response to DMOG. Reporter assay analysis demonstrates reduced HIF-1α transcriptional activity in cells overexpressing SUMO-1 and SUMO-2/3, whereas pretreatment with TAK-981 increased HIF-1α transcriptional activity in response to DMOG. In addition, HIF-1α nuclear accumulation was decreased in cells overexpressing SUMO-1. Importantly, we showed that HIF-1α is not directly SUMOylated, but that SUMOylation affects HIF-1α stability and activity indirectly. Taken together, our results indicate that SUMOylation indirectly suppresses HIF-1α protein stability, transcriptional activity, and nuclear accumulation in intestinal epithelial cells.

Most multicellular organisms require a continuous supply of molecular oxygen (O_2_) for the survival and maintenance of cellular bioenergetic homeostasis. Hypoxia arises when the cellular oxygen demand exceeds the vascular supply. On a cellular level, tissue adaptation to hypoxia is primarily controlled by the hypoxia-inducible factor (HIF) signaling pathway. HIFs are heterodimeric transcription factors that regulate the cellular response to hypoxia by inducing the expression of over 400 hypoxia-dependent genes involved in adaptive processes, such as glycolysis, erythropoiesis, and cell survival, consequently, normalizing oxygen homeostasis in hypoxic tissues (1). In normoxia, most O_2_ is used by the mitochondria in the generation of ATP with the remaining “spare” O_2_ facilitating hydroxylation of HIF by prolyl hydroxylase domain enzymes and factor-inhibiting HIF, leading to ubiquitin binding by the von Hippel–Lindau E3 ubiquitin ligase complex (2). This leads to proteasomal degradation and transcriptional repression of HIF. In hypoxia, virtually all O_2_ is used by the mitochondria resulting in hydroxylase inhibition. Stabilized HIF-1α dimerizes with HIF-1β, thereby forming a heterodimer that binds to hypoxia-response elements (HREs) and CREB-binding protein/p300, leading to increased transcriptional expression of HIF-dependent genes, thus driving the cellular transcriptional response to hypoxia (1, 3, 4).

Oxygen-dependent regulation of HIF-1α by prolyl hydroxylases and the von Hippel–Lindau E3 ligase complex has been well characterized in multiple cell types (5, 6). Alternative oxygen-independent post-translational modifications, including acetylation, phosphorylation, and SUMOylation, have been reported to affect HIF-1α stability and activity, although the functional impact of such modifications is less clear (7). SUMOylation, the attachment of small ubiquitin-like modifier (SUMO) proteins to a target protein, affects multiple biologic processes, including DNA repair, transcription, and cell cycle (8, 9). There are four major SUMO isoforms (SUMO1–4) ubiquitously expressed in mammalian cells (10). While SUMO-2 and SUMO-3 share similar structure and differ from SUMO-1, all three isoforms modify a distinct yet inter-related group of proteins (7). SUMO conjugation to a target protein is a three-step process facilitated by the actions of SUMO-specific enzymes, including SUMO-activating enzymes E1 and E2, SUMO-conjugating enzyme E2 (Ubc9), and a SUMO E3-ligase (11, 12, 13). Protein SUMOylation can be then reversed by SUMO sentrin-specific proteases known as SENPs that cleave isopeptide bonds between substrate and SUMO proteins (14). Recent studies demonstrated that SUMOylation is sensitive to hypoxia and has been reported to regulate the HIF pathway (7, 15, 16). However, the impact of SUMO modifications on the stability and activity of HIF-1α remains highly controversial.

In this study, we elucidated the effects of SUMOylation on the expression pattern of HIF-1α in response to hydroxylase inhibitor dimethyloxalylglycine (DMOG) in intestinal epithelium. Previous work from our group has shown HIF-1α to be highly protective in models of inflammatory bowel disease (17, 18, 19). Our data suggest that SUMO-1, SUMO-2, and SUMO-3 isoforms indirectly suppress HIF-1α, whereas pharmacologic inhibition of the SUMO pathway with TAK-981 upregulates HIF-1α post-translationally in intestinal epithelial cells.

## Results

### TAK-981 promotes dose- and time-dependent decreases in global SUMO-1 and SUMO-2/3 modification in intestinal epithelial cells

**Figure 1:**
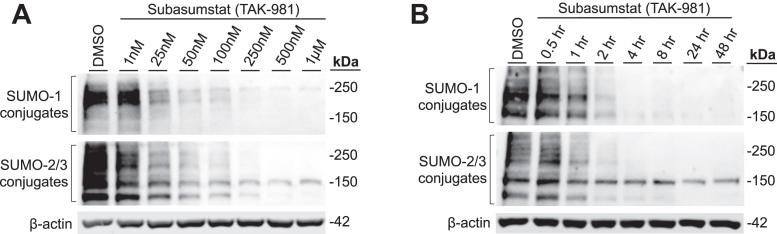
**Inhibition of global SUMO-1 and SUMO-2/3 modifications with TAK-981 in intestinal epithelial cells.***A*, Western blot analysis of SUMO-1 and SUMO-2/3 conjugates in Caco-2 cells treated with DMSO (vehicle) or increasing concentrations of TAK-981 for 4 h. *B*, Western blot analysis of SUMO-1 and SUMO-2/3 conjugates in Caco-2 cells stimulated with DMSO (vehicle) or TAK-981 (100 nM) for increasing time points. β-actin was used as a loading control. Representative blots of n = 3 independent experiments are shown. DMSO, dimethyl sulfoxide; SUMO, small ubiquitin-like modifier.

We first demonstrated that the novel pharmacological SUMO inhibitor TAK-981 inhibits the SUMO pathway in Caco-2 intestinal epithelial cells in a dose- and time-dependent manner by analyzing global SUMO-1 and SUMO-2/3 modifications (Fig. 1). TAK-981 blocks SUMO protein functionality by inhibiting the SUMO-activating enzyme within the first step of E1 SUMO enzymatic cascade (20). We found that treatment of Caco-2 cells with increasing concentrations of TAK-981 (1 nM–1 μM) for 4 h resulted in a dose-dependent decrease in global SUMO-1 and SUMO-2/3 conjugation, thus reflecting responsiveness of these cells to pharmacologic inhibition of the SUMO enzymatic cascade (Fig. 1*A*). Downregulation of SUMO-1 and SUMO-2/3 modifications was a later event, with their expression fully inhibited at 4 h after treatment with 100 nM of TAK-981 (Fig. 1*B*). A dose of 100 nM of TAK-981 produced substantial inhibition of the SUMO pathway after 4 to 6 h, thus we selected this dosage and time points for further experiments.

### TAK-981 enhances HIF-1α protein expression

**Figure 2:**
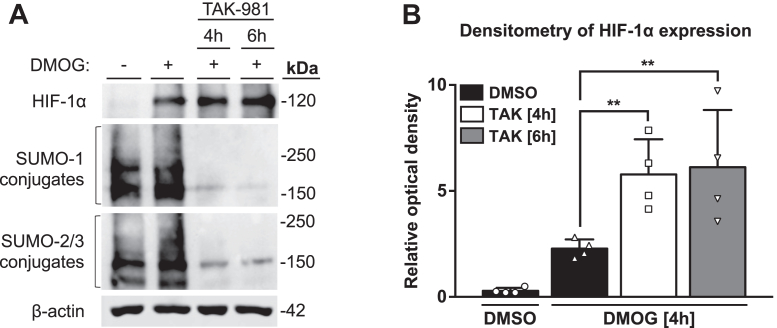
**Effects of TAK-981 on HIF-1α stabilization by DMOG.***A*, Western blot analysis of HIF-1α, SUMO-1, and SUMO-2/3 conjugates in Caco-2 cells treated with DMOG (1 mM) for 4 h, simultaneously treated with TAK-981 (100 nM) and DMOG for 4 h or pretreated with TAK-981 for 2 h, and then treated with DMOG for 4 h. DMSO was used as a vehicle control. β-actin was used as a loading control. Representative blots of n = 4 independent experiments are shown. *B*, densitometric analysis of HIF-1α blots normalized by the β-actin expression from *A* using Image Studio Lite. Data presented as the mean ± SD (n = 4). DMOG, dimethyloxalylglycine; DMSO, dimethyl sulfoxide; HIF, hypoxia-inducible factor; SUMO, small ubiquitin-like modifier.

Having demonstrated the inhibitory effects of TAK-981 on the SUMO pathway, we next investigated the impact of TAK-981 on HIF-1α protein levels. The pan-hydroxylase inhibitor DMOG was used to stabilize the HIF pathway in Caco-2 cells. To determine whether SUMOylation affects HIF-1α expression, cells were simultaneously treated with TAK-981 and DMOG for 4 h or pretreated with TAK-981 for 2 h prior to treatment with DMOG for 4 h. We found that inhibition of the SUMO pathway with TAK-981 enhanced HIF-1α protein expression in response to DMOG (Fig. 2*A*). Densitometric analysis of HIF-1α expression confirmed upregulation of HIF-1α protein levels in cells treated with a combination of TAK-981 and DMOG (Fig. 2*B*). Western blot together with densitometric analysis validate that pharmacologic inhibition of SUMOylation with TAK-981 enhances sensitivity of Caco-2 cells to DMOG by elevating HIF-1α protein levels. Together, our results indicate that SUMOylation is a negative regulator of HIF-1α stability in intestinal epithelial cells.

### SUMO-1 and SUMO-2/3 overexpression suppresses HIF-1α protein levels

**Figure 3:**
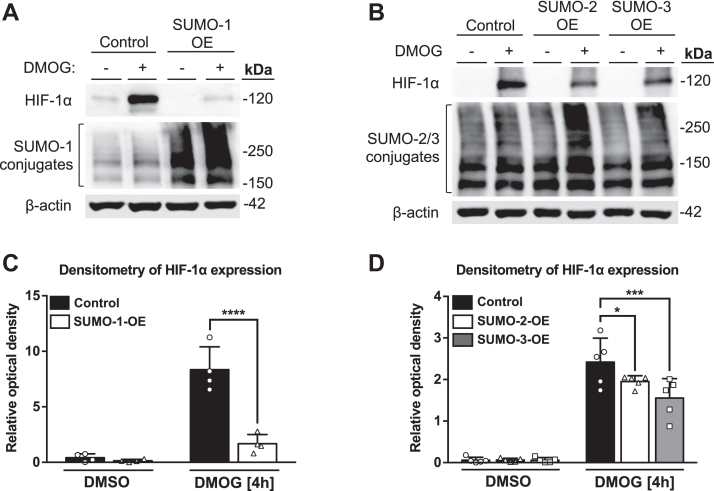
**Overexpression of SUMO isoforms suppresses HIF-1α protein levels.***A*, Western blot analysis of HIF-1α protein and SUMO-1 conjugates in Caco-2 cells transfected with SUMO-1 overexpressing plasmid (500 ng) for 48 h prior to stimulation with DMSO (vehicle) or DMOG (1 mM) for 4 h. Representative blots of n = 4 independent experiments are shown. *B*, Western blot analysis of HIF-1α protein and SUMO-2/3 conjugates in Caco-2 cells transfected with SUMO-2 or SUMO-3 overexpressing plasmids (500 ng each) for 48 h and then treated with DMSO (vehicle) or DMOG for 4 h. Representative blots of n = 5 independent experiments are shown. β-actin was used as a loading control. *C*, densitometric analysis of HIF-1α blots normalized by the β-actin expression from *A* using Image Studio Lite (n = 4). *D*, densitometric analysis of HIF-1α blots normalized by the β-actin expression from *B* using Image Studio Lite (n = 5). Data presented as the mean ± SD. DMOG, dimethyloxalylglycine; DMSO, dimethyl sulfoxide; HIF, hypoxia-inducible factor; SUMO, small ubiquitin-like modifier.

Having demonstrated that pharmacological inhibition of the SUMO pathway enhanced HIF-1α expression, we next evaluated the effects of SUMO-1, SUMO-2, and SUMO-3 overexpression on HIF-1α protein levels in response to DMOG. To determine whether SUMO isoforms affect HIF-1α expression, we overexpressed SUMO-1, SUMO-2, or SUMO-3 protein for 48 h and then treated cells with DMOG. SUMO-1 overexpression resulted in a decrease in HIF-1α protein expression in response to DMOG, which was correlated with an enhanced level of SUMO-1 modifications (Fig. 3*A*). Furthermore, SUMO-2 and SUMO-3 overexpression also promoted a decrease in HIF-1α protein levels; however, the effects of overexpressed SUMO-2/3 isoforms on global SUMO-2/3 modifications were less pronounced (Fig. 3*B*). Densitometric analysis of HIF-1α expression further confirmed reduced HIF-1α protein expression in cells overexpressing various SUMO isoforms (Fig. 3, *C* and *D*). Western blot together with densitometric analysis validate that SUMO-1, SUMO-2, and SUMO-3 suppress DMOG-activated HIF-1α protein expression in Caco-2 cells.

### SUMOylation decreases HIF-1α transcriptional activity

**Figure 4:**
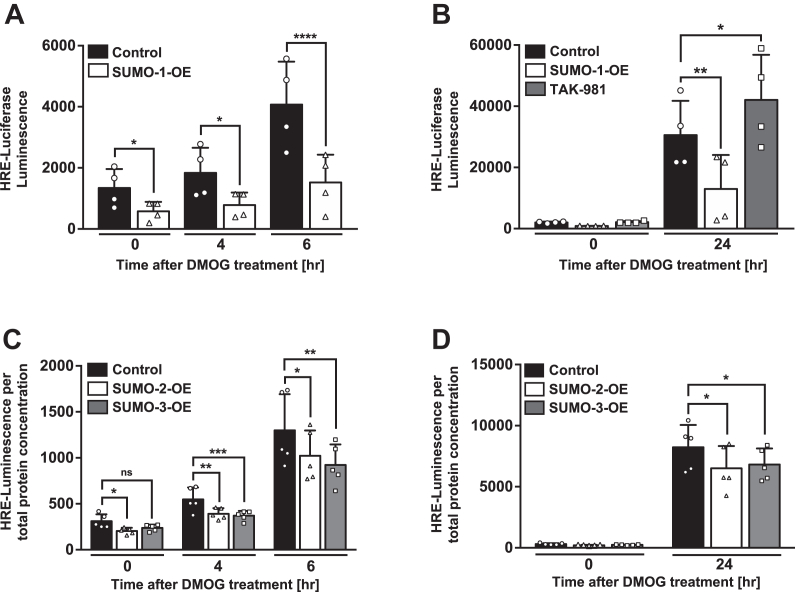
**SUMOylation regulates HIF-1α transcriptional activity in Caco-2 cells.***A*, cells were transfected with HRE Gaussia Luciferase construct (750 ng) and SUMO-1 overexpressing plasmid (500 ng) for 48 h and then treated with DMSO (vehicle, 0 h) or DMOG (1 mM) for 6 h. HRE luciferase activity was measured in the cell-growth media after 0, 4, and 6 h of DMOG treatment. *B*, Caco-2 cells were transfected with HRE luciferase construct and SUMO-1 overexpressing plasmid for 48 h or transfected with HRE Luciferase construct for 48 h, prior to treatment with TAK-981 (100 nM) for 2 h. Cells were then stimulated with DMSO or DMOG for 24 h. HRE luciferase activity was determined in the cell-growth media after 24 h of DMOG treatment by HRE Gaussia luciferase assay (n = 4, ∗∗∗∗*p* < 0.0001, ∗∗*p* < 0.01, ∗*p* < 0.05). *C* and *D*, cells were transfected with HRE Luciferase construct, SUMO-2 and SUMO-3 overexpressing plasmids (500 ng each) for 48 h, and then treated with DMSO or DMOG for 24 h. HRE luciferase activity was measured in the cell-growth media after 0, 4, 6, and 24 h of DMOG treatment (n = 5, ∗∗∗*p* < 0.0005, ∗∗*p* < 0.01, ∗*p* < 0.05; ns, nonstatistically significant). Data presented as the mean ± SD. DMOG, dimethyloxalylglycine; DMSO, dimethyl sulfoxide; HIF, hypoxia-inducible factor; HRE, hypoxia-response element; SUMO, small ubiquitin-like modifier.

Having demonstrated a negative effect of SUMOylation on HIF-1α protein expression, we next evaluated the impact of SUMO-1 and SUMO-2/3 overexpression and TAK-981 on HIF-1α activity at the transcriptional level in intestinal epithelium. Using an HRE Gaussia Luciferase reporter system, we demonstrated that stimulation of cells with DMOG resulted in time-dependent increase in HRE activity, which was dependent upon functional HIF-1α transcriptional activation (Fig. 4). Overexpression of SUMO-1 in Caco-2 cells resulted in significant downregulation of HIF-1α transcriptional activity in response to DMOG at early time points (Fig. 4*A*). Interestingly, before DMOG administration to the cells (0 h time point), basal HIF-1α transcriptional activity was decreased because of SUMO-1 overexpression. In contrast, pretreatment with TAK-981 had no effect on HIF-1α transcriptional activity in cells stimulated with DMOG (0–6 h time points, Fig. S3). By analyzing HIF-1α transcriptional activity after 24 h of DMOG treatment, we found that Caco-2 cells overexpressing SUMO-1 had significantly reduced HIF-1α transcriptional activity, whereas administration of TAK-981 resulted in enhanced HIF-1α activity (Fig. 4*B*). Moreover, we observed similar result when cells were overexpressing SUMO-2 and SUMO-3 isoforms. HIF-1α transcriptional activity was significantly reduced at early time points (0–6 h) and after 24 h of DMOG treatment because of SUMO-2 and SUMO-3 overexpression (Fig. 4, *C* and *D*). Altogether, these data indicate that SUMO-1, SUMO-2, and SUMO-3 reduce HIF-1α transcriptional activity in Caco-2 cells before stimulation and after stimulation with DMOG, whereas inhibition of the SUMO pathway with TAK-981 enhances HIF-1α transcriptional activity only after 24 h of DMOG treatment.

### SUMO-1 regulates HIF-1α nuclear accumulation

**Figure 5:**
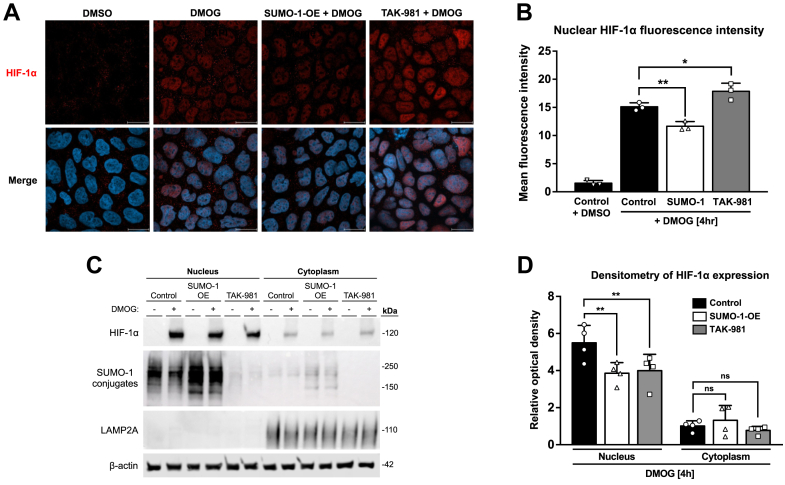
**Effects of SUMOylation on HIF-1α nuclear accumulation in intestinal epithelial cells.***A*, immunofluorescence with laser-scanning confocal microscope images of HIF-1α (*red*) and nuclei (DAPI, *blue*) in Caco-2 cell treated with either DMSO (vehicle) or DMOG (1 mM) for 4 h, transfected with SUMO-1 overexpressing plasmid (500 ng) for 48 h and treated with DMOG for 4 h, or pretreated with TAK-981 (100 nM) for 2 h and then stimulated with DMOG for 4 h. Cells were fixed with 4% paraformaldehyde and stained with rabbit anti-HIF-1α (*red*) and DAPI nuclear staining (*blue*). Images were captured using Zeiss LSM 800 laser-scanning confocal microscope with 63× oil-immersion objective, n = 3. Scale bar represents 20 μm. *B*, quantification of nuclear HIF-1α fluorescence intensity in Caco-2 cells using ImageJ. Data presented as the mean ± SD for n = 5 different fields of view for n = 3 independent experiments (∗∗*p* < 0.01 and ∗*p* < 0.05). *C*, Western blot analysis of HIF-1α and SUMO-1 conjugates from nuclear and cytoplasmic fractions of Caco-2 cells treated with DMSO (vehicle) or DMOG (1 mM) for 4 h, transfected with SUMO-1 overexpressing plasmid (500 ng) for 48 h and treated with DMOG for 4 h, or pretreated with TAK-981 (100 nM) for 2 h prior to treatment with DMOG for 4 h. LAMP2A was used as a cytoplasmic marker. β-actin was used as a loading control. Representative blots of n = 4 independent experiments are shown. *D*, densitometric analysis of nuclear and cytoplasmic HIF-1α blots normalized by the β-actin expression from *C* using Image Studio Lite. Data presented as the mean ± SD (∗∗*p* < 0.01). DAPI, 4′,6-diamidino-2-phenylindole; DMOG, dimethyloxalylglycine; DMSO, dimethyl sulfoxide; HIF, hypoxia-inducible factor; SUMO, small ubiquitin-like modifier.

Having established that SUMO-1 isoform promoted the strongest decrease in HIF-1α protein expression and transcriptional activity when compared with SUMO-2/3 isoforms, we next investigated whether the changes in HIF-1α in the presence of overexpressed SUMO-1 and TAK-981 were due to the effects of SUMOylation on nuclear accumulation of HIF-1α. By using confocal immunofluorescence microscopy, we demonstrated accumulation of stabilized HIF-1α in the nuclei in Caco-2 cells treated with DMOG when compared with those treated with dimethyl sulfoxide (Fig. 5*A*). We found that cells overexpressing SUMO-1 displayed lower nuclear levels of accumulated HIF-1α, whereas inhibition of the SUMO pathway with TAK-981 enhanced nuclear stabilization of HIF-1α (Fig. 5*A*). By analyzing the mean fluorescent intensity of DMOG-induced HIF-1α nuclear levels, it became evident that SUMO-1 overexpression significantly reduced it, whereas TAK-981 upregulated HIF-1α nuclear accumulation (Fig. 5*B*). We further investigated the effect of SUMOylation on nuclear and cytoplasmic protein levels of HIF-1α using subcellular fractionation. We found that SUMO-1 overexpression reduced nuclear protein levels of HIF-1α (Fig. 5, *C* and *D*). However, pretreatment of cells with TAK-981 also reduced nuclear levels of HIF-1α. We also observed that global protein SUMOylation with SUMO-1 mainly occurs in the nucleus. Our results suggest that SUMO-1 controls DMOG-induced HIF-1α nucleocytoplasmic shuttling, whereas inhibition of the SUMO pathway with TAK-981 seems to have a more complex effect on HIF-1α localization in intestinal epithelium.

### HIF-1α is not directly SUMOylated in intestinal epithelial cells

**Figure 6:**
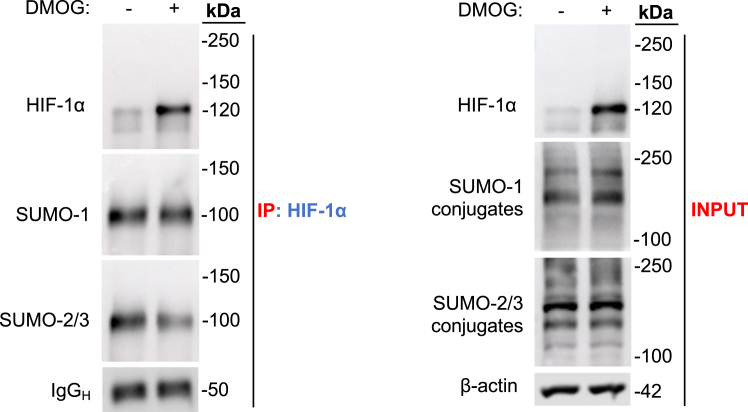
**HIF-1α is not directly SUMOylated in intestinal epithelial cells.** Western blot analysis of HIF-1α, SUMO-1, and SUMO-2/3 conjugate expression following coimmunoprecipitation of HIF-1α from whole-cell lysates of Caco-2 cells treated with DMSO (vehicle) or DMOG (1 mM) for 4 h. Input = 10% protein concentration of total immunoprecipitation. β-actin was used as a loading control. Representative blots of n = 3 independent experiments are shown. DMOG, dimethyloxalylglycine; DMSO, dimethyl sulfoxide; HIF, hypoxia-inducible factor; SUMO, small ubiquitin-like modifier.

Finally, our previous findings demonstrate that SUMOylation significantly influences HIF-1α activity and stability in intestinal epithelial cells. Therefore, we investigated whether HIF-1α could be directly SUMOylated. To do this, we coimmunoprecipitated HIF-1α from whole-cell lysates of Caco-2 cells treated with DMOG for 4 h. The expression of HIF-1α, SUMO-1, and SUMO-2/3 proteins was determined in coimmunoprecipitation (co-IP) and input samples by Western blot. We did not detect direct SUMOylation of HIF-1α in our system. Upon analyzing the coimmunoprecipitated HIF-1α samples, we observed the presence of SUMO-1 and SUMO-2/3 modified proteins, as demonstrated by bands around 100 kDa (Fig. 6). However, considering that the molecular weight of HIF-1α is approximately 120 kDa, higher molecular weight SUMO conjugates bound to HIF-1α should be present above 120 kDa. Our findings suggest that HIF-1α is not directly SUMOylated in our system, as we did not detect SUMO-modified HIF-1α. This indicates that there may be other lower molecular weight HIF-interacting proteins that undergo SUMOylation, suggesting their potential role in controlling HIF-1α stability prior to its stabilization with DMOG.

## Discussion

Considering the significance of SUMOylation in regulating biological functions in various pathophysiological conditions, a number of small-molecule inhibitors targeting SUMOylation have been suggested as potential therapeutic opportunities for multiple diseases (21, 22). To better understand the effects of SUMOylation on HIF-1α, we took a novel pharmacological approach to inhibit protein SUMOylation in our system. Recent studies have demonstrated inhibition of global SUMO-1 and SUMO-2/3 modifications by using a selective inhibitor of the SUMO-enzymatic cascade, subasumstat (TAK-981) (20). The impact of TAK-981 has recently been evaluated in patients with lymphoma and metastatic solid tumors in phase 1/2 clinical trials, highlighting the importance of implementing SUMO inhibitors in the treatment of multiple diseases (23). In this study, we confirmed that treatment of Caco-2 cells with TAK-981 resulted in dose- and time-dependent reduction in global SUMO-1 and SUMO-2/3 modifications. To the best of our knowledge, this is the first study that demonstrates the effects of pharmacologic inhibition of the SUMO pathway using precisely optimized concentrations and treatment durations of TAK-981 in Caco-2 cells. Therefore, by using TAK-981, we can further investigate the effects of SUMOylation on HIF-1α in intestinal epithelial cells.

The HIF pathway has a strong impact on epithelial cell function and development *via* activation of protective transcriptional responses (24). Stabilization of HIF-1α by DMOG is a crucial role in both *in vivo* and *in vitro* models of inflammation, as it prevents epithelial cell apoptosis and improves barrier function (19, 25). In this study, we took the novel strategy of combining TAK-981 and DMOG in an attempt to change the sensitivity of Caco-2 cells to hydroxylase inhibition *via* activation of HIF-1α. Therefore, we investigated the impact of the SUMO pathway inhibition with TAK-981 on HIF-1α protein expression in response to DMOG. We discovered that using TAK-981 to inhibit protein SUMOylation resulted in increased levels of HIF-1α protein. In addition, we found a direct correlation between the duration of TAK-981 treatment and the expression of HIF-1α. Moreover, TAK-981 did not affect basal levels of HIF-1α before its activation with DMOG (Fig. S2). Our data suggest that pharmacologic modulation of the SUMO pathway with TAK-981 positively regulates HIF-1α in our system, thereby highlighting the potential benefit of using TAK-981 to enhance activity of hydroxylase inhibitors in the treatment of intestinal disorders.

For a target protein to be SUMOylated, it must undergo a three-step enzymatic cascade reaction similar to ubiquitination. SUMOylation influences various aspects of protein function, including protein–protein interactions, stability, and biological activity. Therefore, after demonstrating that the inhibition of SUMOylation enhances stabilization of HIF-1α, we further investigated the impact of overexpressing major SUMO isoforms, including SUMO-1, SUMO-2, and SUMO-3, on HIF-1α protein expression in response to DMOG. Recent studies have revealed diverse effects of SUMO isoforms on HIF-1α function and stability. SUMO-1 has been found to increase HIF-1α protein levels by SUMOylating it at two lysine residues in HeLa and human embryonic kidney 293T cells (26). SUMO-1 knockdown decreased HIF-1α protein levels, whereas SUMO-1 overexpression increased HIF-1α protein expression in rat pulmonary arterial smooth muscle cells (27). Conversely to these findings, decreased HIF-1α protein levels were linked to an increase in global SUMO-1 and SUMO-2/3 modifications in HCT116 cells (28). Our findings reveal that SUMO-1 overexpression decreases HIF-1α protein levels in response to DMOG treatment in Caco-2 cells. Furthermore, despite the 95% identity shared between SUMO-2 and SUMO-3 isoforms, these variants can exert different mechanistic impacts on their target proteins (29). We demonstrated that overexpressing SUMO-2 and SUMO-3 isoforms led to a decrease in HIF-1α protein levels upon DMOG treatment. In addition, we demonstrated that overexpressing SUMO-1, SUMO-2, and SUMO-3 or treating cells with TAK-981 did not affect HIF-1α mRNA expression (Fig. S1). Taken together, our research suggests that all three SUMO isoforms play roles in modulating HIF-1α protein stability without altering transcriptional activation of HIF-1α, indicating post-translational modulation of HIF-1α; however, it remains unclear whether SUMO-1/2/3 directly interact with and modify HIF-1α or do so indirectly.

Under hypoxic conditions, stabilized HIF-1α forms HIF-αβ heterodimers that bind to HREs and CREB-binding protein/p300 within a nucleus, thus creating a transcriptionally active complex that promotes elevated expression of HIF-dependent adaptive genes (30). Therefore, when focusing our attention on the transcriptional levels of HIF-1α, it was further evident that overexpressing SUMO-1, SUMO-2, and SUMO-3 led to a significant decrease in HRE activity in response to DMOG, which was dependent upon functional HIF-1α transcriptional activation. Moreover, SUMO-1 overexpression resulted in a reduction of HIF-1α transcriptional activity even before DMOG was administered to the cells. A few studies reported the same observation where HIF-1α modified by SUMO displayed reduced transcriptional activity in HeLa cells (31). Furthermore, SUMOylation of prolyl hydroxylase domain 3 with SUMO-2/3 resulted in decreased HIF-1α transcriptional activity (32). However, this contrasts with previous reports that showed an increase in HIF-1α transcriptional activity when overexpressing SUMO-1 or SUMO-2 isoforms (26, 33). Finally, we observed that while inhibition of SUMOylation with TAK-981 enhanced HIF-1α transcriptional activity after 24 h of DMOG treatment, it did not impact HIF-1α at earlier time points (Fig. S3). As of now, there are no studies that investigated the effects of TAK-981 on HIF-1α stability and activity. Mechanisms through which SUMO regulates HIF-1α could be an intriguing area for future research.

Modification of cytoplasmic proteins by SUMO involves changes in their nucleocytoplasmic shuttling and nuclear accumulation depending on the target protein (34). For example, SUMOylation of p53 enhances its nuclear transport from the cytosol (35). Mitogen/extracellular signal–regulated kinase kinase-5–dependent activation of extracellular signal–regulated kinase 5 increased extracellular signal–regulated kinase 5 nuclear translocation by SUMOylating it with SUMO-2 (36). On the contrary, mutation within SUMO-1 sites abrogated insulin-like growth factor 1 receptor nuclear translocation (37). In the absence of hydroxylation, HIF-1α stabilizes in the cytoplasm and translocates to the nucleus (1). Supporting this, we found that SUMO-1 overexpression reduced the accumulation of stabilized HIF-1α in the nuclei, whereas the inhibition of SUMOylation with TAK-981 amplifies the nuclear stabilization of HIF-1α in cells treated with DMOG. To further validate this, using subcellular fractionation, we found that SUMO-1 overexpression decreased nuclear HIF-1α protein expression. Pretreatment with TAK-981 also decreased DMOG-induced nuclear HIF-1α protein expression. Therefore, these results suggest that SUMO-1 controls nucleocytoplasmic shuttling and nuclear accumulation of HIF-1α in intestinal epithelial cells.

Having generated a significant body of evidence that strongly supports the involvement of SUMOylation in the regulation of HIF-1α at various levels, the last critical question that needs to be clarified is whether HIF-1α is directly SUMOylated in intestinal epithelial cells. SUMOylation of target proteins usually occurs on a lysine residue (38). Thus, SUMOylation of HIF-1α with SUMO-1 is known to occur at two lysine residues, Lys391 and Lys477 (26). Moreover, other studies confirmed that HIF-1α can be directly SUMOylated with SUMO-1 and SUMO-2/3 under both normoxic and hypoxic conditions (27, 31, 39). In our study, we found no evidence for direct HIF-1α SUMOylation in this system. Interestingly, we detected interaction of SUMO-1 and SUMO-2/3 isoforms with a protein coimmunoprecipitated with HIF-1α; however, these interactions were detected around 100 kDa. Our results indicate that HIF-1α is not directly SUMOylated in intestinal epithelial cells, suggesting the presence of HIF-regulating protein that undergoes SUMOylation.

In conclusion, to date, the role of SUMOylation in controlling HIF-1α protein stability has been unclear with respect to consequences for HIF signaling. For the first time, our study demonstrated the negative regulatory function of SUMOylation in controlling HIF-1α stability, transcriptional activity, and nucleocytoplasmic shuttling in intestinal epithelial cells. Notably, all three SUMO isoforms, SUMO-1/2/3, can mediate this effect. We also discovered that inhibition of the SUMO pathway with TAK-981 can reverse these effects, thereby enhancing the protective DMOG-induced HIF responses in these cells. Importantly, while many studies have suggested direct SUMOylation of HIF-1α, our data indicate that HIF-1α is not directly SUMOylated in Caco-2 cells. We conclude that SUMOylation plays an important role in shaping the nature of the adaptive HIF response to hypoxia in intestinal epithelium by suppressing HIF-1α, thus emphasizing the importance of investigating the involvement of SUMOylation in the regulation of the HIF pathway.

## Experimental procedures

### Cell culture

Human colonic epithelial Caco-2 cells (American Type Culture Collection HTB-37) were cultured in Dulbecco’s modified Eagle's medium (Lonza) containing 4.5 g/l glucose. Media were supplemented with 10% fetal bovine serum (Gibco), 1% penicillin/streptomycin antibiotic solution (100 units/ml; Gibco), and 1% MEM nonessential amino acid solution (Gibco). Cells were maintained in an incubator at 37 °C, 21% O_2,_ and 5% CO_2_.

### Compound treatments

Dimethyl sulfoxide was used as vehicle control. The SUMOylation enzymatic cascade inhibitor subasumstat (TAK-981) was obtained from MedChemExpress (HY-111789). The pan-hydroxylase inhibitor DMOG was purchased from Cayman Chemical (catalog no.: 71210). Reagent doses and treatment durations are listed in the figure legends.

### Plasmid transfection

Human SUMO-1, SUMO-2, and SUMO-3 complementary DNA (cDNA) plasmids were purchased from OriGene (RC221677, RC224336, and RC200241, respectively). HRE-Gaussia Luciferase reporter construct was provided by Dr Alex Cheong (Aston University). Caco-2 cells were grown to 30% confluency. Before transfection, the media were replaced with penicillin/streptomycin-free Dulbecco’s modified Eagle's medium containing 10% fetal bovine serum and 1% nonessential amino acid. Cells were transfected with 500 ng of SUMO-1, SUMO-2, or SUMO-3 plasmid and/or 750 ng of HRE-GLuc plasmid for 48 h using Lipofectamine 2000 (Invitrogen; catalog no.: 11668027) and Opti-MEM (Gibco). After transfection, the cells were treated as desired.

### Subcellular fractionation

To isolate cytoplasmic and nuclear fractions, cell-culture media were removed, and cells were washed with ice-cold PBS. Cells were lysed using cytoplasmic lysis buffer containing 10 mM Hepes (pH 8), 1 mM MgCl_2_, 10 mM KCl, 200 mM sucrose, 1% NP-40 supplemented with 1× protease inhibitor cocktail (PIC; Sigma) and 40 mM *N*-ethylmaleimide (NEM; Sigma), incubated on ice for 10 min, and centrifuged at 12,000 rpm for 5 min at 4 °C. Supernatant containing cytoplasmic proteins was collected, and the pellet was resuspended in nuclear lysis buffer containing 20 mM Hepes (pH 8), 1.5 mM MgCl_2_, 420 mM NaCl, 0.2 mM EDTA, 0.5 mM DTT, 25% glycerol supplemented with 1× PIC and 40 mM NEM. Samples were incubated for 30 min on ice with gentle agitation and then centrifuged at 14,000 rpm for 5 min at 4 °C. The collected supernatant contained nuclear fraction.

### Immunoblotting

Whole-cell protein lysates were generated by lysing cells in radioimmunoprecipitation assay buffer (Merck) containing 50 mM Tris–HCl, pH 7.4, 150 mM NaCl, 1% NP-40, and 10 μM EDTA and supplemented with 1× PIC and 40 mM NEM. Proteins were denatured by boiling samples at 95 °C for 5 min in 1× SDS loading buffer containing 5% mercaptoethanol, resolved in 8% or 10% SDS-polyacrylamide gels by SDS-PAGE and then transferred onto 0.45 μm nitrocellulose membranes by a wet transfer system (Bio-Rad). The membranes were blocked in 5% milk for 1 h and incubated with primary antibodies overnight at 4 °C. Western blot analysis was performed using the following antibodies: HIF-1α (D1S7W) XP rabbit monoclonal antibody #36169, SUMO-1 #4930, SUMO-2/3 (18H8) rabbit monoclonal antibody #4971, anti-rabbit immunoglobulin G (IgG) horseradish peroxidase–linked #7074 (all 1:1000 dilution; Cell Signaling), anti-β-actin A5441 (1:5000 dilution; Sigma), and DyLight 680 Goat Antimouse IgG #35519 (1:5000 dilution; Invitrogen). Enhanced chemiluminescence WesternBright horseradish peroxidase substrate (Advansta) was used for signal immunodetection by Fusion FX Spectra Imager (Vilber Lourmat). Fluorescent signal was detected by Odyssey CLx Imager (Li-Cor Biosciences). Quantification of protein band intensity was analyzed using Image Studio Lite software (Li-Cor Biosciences) and normalized to the density of the β-actin band.

### Co-IP

Whole-cell protein lysates were generated as previously described and normalized to 1 mg of protein. Normalized protein lysates were incubated with a primary antibody against HIF-1α (1:75 dilution; Cell Signaling; catalog no.: 36169) overnight at 4 °C with end-to-end rotation. Input samples were normalized to 10% of the co-IP samples by adding 5× sample buffer, beta-mercaptoethanol, and distilled water, following by boiling at 95 °C for 5 min. To isolate the antibody-bound proteins, 20 μl of Protein A Agarose Beads (Cell Signaling; catalog no.: 9863) were added to each sample and incubated for 4 h at 4 °C with end-to-end rotation. Agarose beads were washed twice with IP wash buffer, containing 100 mM Tris–HCl, 300 mM NaCl, and 10 mM MgCl_2_, to remove nonspecific interactors. For Western blotting, 1× NuPAGE LDS sample buffer (ThermoFisher; catalog no.: NP0007) was added to samples, and they were then heated at 95 °C for 10 min with 300 rpm rotation to elute antibody-bound proteins. Samples were then centrifuged at 2000 rpm for 1 min at 4 °C to precipitate the beads. Supernatant was collected, and 100 mM DTT was added to each sample and boiled at 95 °C for 5 min. Co-IP protein samples were resolved by SDS-PAGE as previously described.

### Immunofluorescence

Caco-2 cells were seeded onto sterilized coverslips coated with 50 μg/ml of poly-d-lysine (Gibco; catalog no.: A38904). After desired treatment, cells were fixed using 4% paraformaldehyde for 10 min and washed with Dulbecco’s PBS without Ca^2+^ and Mg (DPBS; Corning). Cells were blocked in 5% donkey serum, 0.3% Triton X-100 (Fisher; catalog no.: 3751) in DPBS for 1 h, and then probed for HIF-1α (1:400 dilution; Cell Signaling, catalog no.: 36169) made up in 0.1% Triton X-100, 0.1 g bovine serum albumin (Fisher; catalog no.: BP9702) in DPBS overnight at 4 °C. The following day, cells were incubated with secondary donkey anti-rabbit IgG Alexa Fluor 568 antibody (1:800 dilution; Invitrogen, catalog no.: A10042) made up in 0.1% Triton X-100 and 0.1 g bovine serum albumin in DPBS for 2 h at 4°C. Cells were then counterstained with 1 μg/ml of 4′,6-diamidino-2-phenylindole nucleic acid stain (1:1000 dilution; Sigma, catalog no.: D9542) in DPBS for 10 min at room temperature. Coverslips were inverted and then mounted onto glass slides using Fluoromount Mounting Media (Sigma; catalog no.: F4680). Slides were imaged using Zeiss LSM800 laser-scanning confocal microscope with 63× oil-immersion objective. HIF-1α nuclear fluorescent intensity was quantified in ImageJ software; National Institutes of Health and the Laboratory for Optical and Computational Instrumentation (University of Wisconsin). “Freehand” selection tool was used to record mean fluorescence intensity by highlighting the nuclear regions in 2D-rendered confocal images acquired from five different fields of view for each biological replicate (n = 3). Nuclei stained with 4′,6-diamidino-2-phenylindole were used as a nuclear localization control. A mean fluorescence intensity was then calculated for each technical and biological replicate.

### HRE-Gaussia luciferase reporter assay

HRE-Gaussia Luciferase reporter system was used to measure HIF-1α transcriptional activity. Caco-2 cells were transfected with 750 ng of HRE-Gluc plasmid for 48 h as previously described. Secreted bioluminescence in the media was collected after each treatment with DMOG. Bioluminescence was quantified in a 96-well plate using the Pierce Gaussia Luciferase Glow Assay Kit (Thermo Scientific; catalog no.: 16161) according to the manufacturer’s protocol. Luminescence was determined using a SpectraMax M3 Microplate Reader set to 140 nm.

### Reverse transcriptase quantitative PCR

RNA was isolated from cells using Trizol TRI Reagent (Sigma; catalog no.: T9424). cDNA was generated from 1 mg of total RNA using Moloney murine leukemia virus reverse transcriptase. Target cDNAs were amplified and quantified using SYBR Green (ThermoFisher; catalog no.: 4367659) on the QuantStudio 7 Flex Real-Time PCR System (Applied Biosystems). Samples were incubated for 1 h and 40 min with the thermal cycle set up to 10 min at 95 °C, 15 s at 95 °C, and 60 s at 60 °C repeated for 40 cycles. Relative fold expression of genes of interest relative to β-actin RNA was analyzed using the δ–δ Ct method. Forward and reverse sequences of SYBR Green oligonucleotide primers were as follows:

HIF-1α_Forward: 5′-TTCCAGTTACGTTCCTCGATCA-3′,

HIF-1α_Reverse: 5′-TTTGAGGACTTGCGCTTTCA-3′,

β-actin_Forward: 5′-CGACAGGATGCAGAAGGAGA-3′,

β-actin_Reverse: 5′-CATCTGCTGGAAGGTGGACA-3′.

### Statistical analysis

Statistical analyses were performed using the GraphPad Prism 7 software (GraphPad Software, Inc). One-way ANOVA followed by Holm–Sidak’s test for multiple comparisons was applied when comparing three or more groups. For all experiments, *p* value < 0.05 was considered to be statistically significant. Results are presented as the mean ± SD.

## Data availability

All data are contained within the article and available from the corresponding author upon reasonable request.

## Supporting information

This article contains supporting information.

## Conflict of interest

The authors declare that they have no conflicts of interest with the contents of this article.
