# Supplementary figures and images for: SUMOylation indirectly suppresses activity of the HIF-1α pathway in intestinal epithelial cells
Source: J Biol Chem. 2023 Sep 22;299(11):105280. doi: 10.1016/j.jbc.2023.105280 (PMC10616383; doi:10.1016/j.jbc.2023.105280)

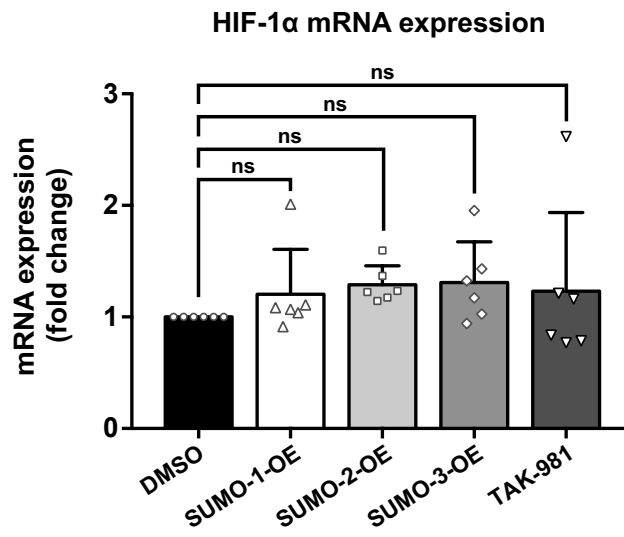

Supplement: Figure S1 [file mmc1.pdf]

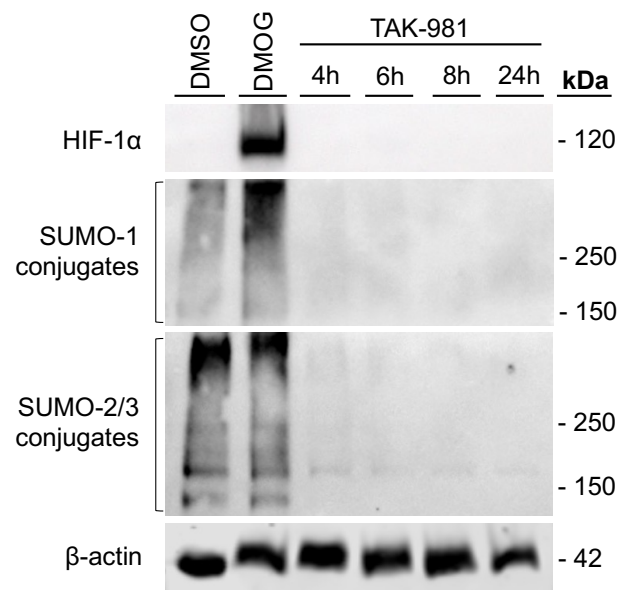

Supplement: Figure S2 [file mmc2.pdf]

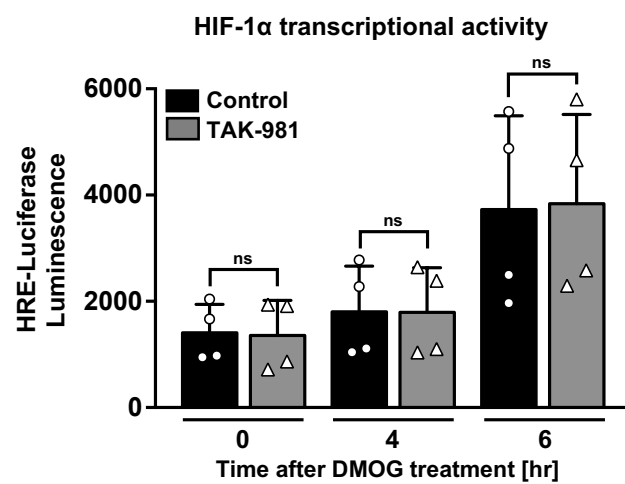

Supplement: Figure S3 [file mmc3.pdf]
